# Supplementary material for: Enzyme-Treated Soybean Meal Enhanced Performance via Improving Immune Response, Intestinal Morphology and Barrier Function of Nursery Pigs in Antibiotic Free Diets
Source: Animals (Basel). 2021 Sep 4;11(9):2600. doi: 10.3390/ani11092600 (PMC8471553; doi:10.3390/ani11092600)
Supplement: Supplementary file 1 [file animals-11-02600-s001.zip › animals-1313329-supplementary.pdf]

animals

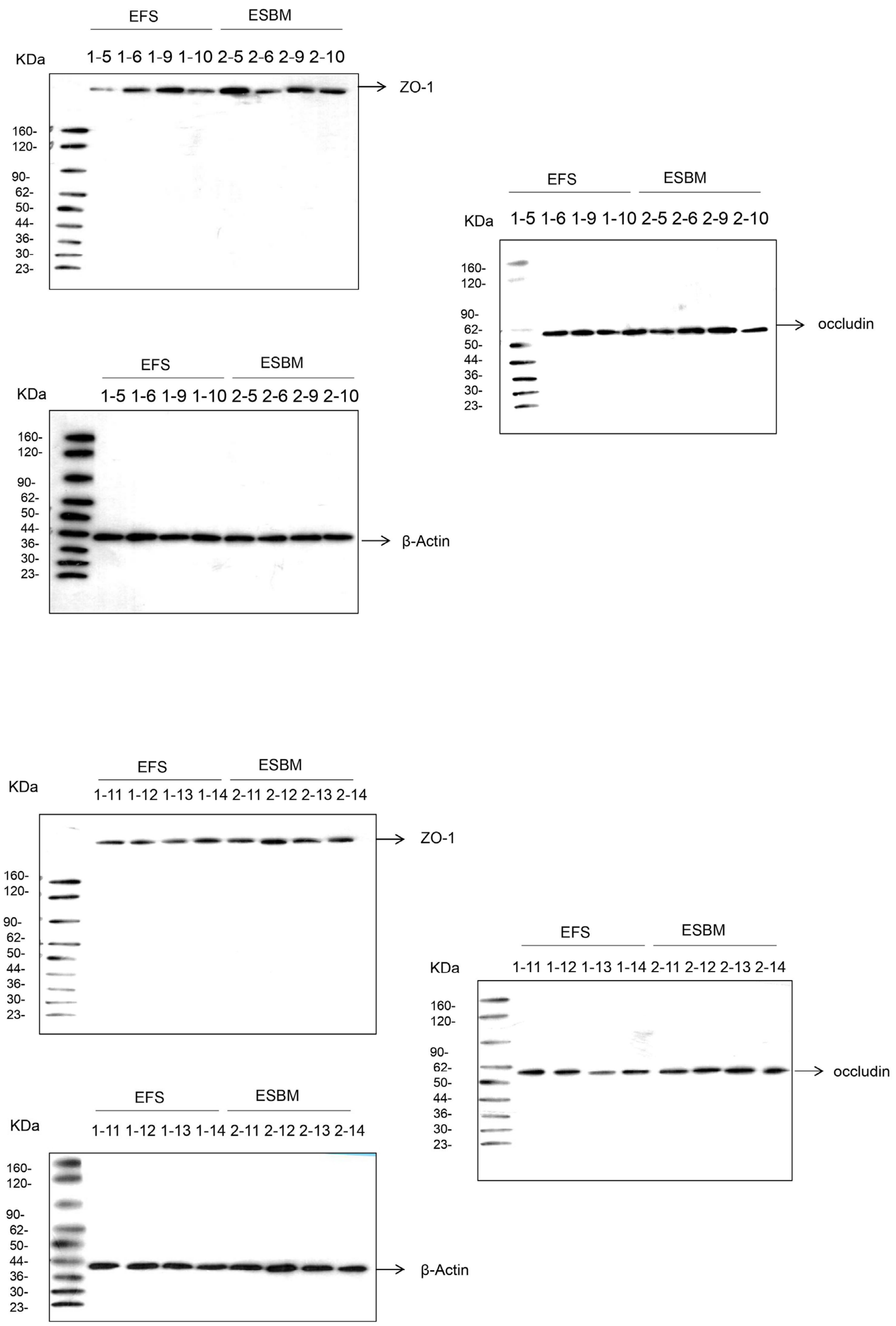

Figure S1. Effects of enzyme-treated soybean meal replacing extruded full-fat soybean on protein expression of ZO-1 and *occludin*. EFS: Extruded full-fat soybean; ESBM: Enzyme-treated SBM.
